# Medical Student Role in the Cardiothoracic Operating Room: A Needs Assessment to Optimize Engagement

**DOI:** 10.1016/j.atssr.2024.09.017

**Published:** 2024-10-16

**Authors:** Kayla N. Laraia, Nathaniel Deboever, Dylan Nieman, Mara B. Antonoff

**Affiliations:** 1 Department of Surgery, Rutgers Robert Wood Johnson Medical School, New Brunswick, New Jersey; 2 Department of Thoracic and Cardiovascular Surgery, University of Texas MD Anderson Cancer Center, Houston, Texas

## Abstract

**Background:**

Focused preparation for the cardiothoracic operating room (CTOR) may optimize intraoperative engagement and medical student interest, but such resources are lacking. We sought to characterize student educational needs in the CTOR to guide resource development.

**Methods:**

A web-based survey targeting cardiothoracic surgeons, trainees, and operating room staff was distributed to identify areas for student improvement in the CTOR. Concepts investigated explored common student mistakes and expectations for knowledge and participation in the CTOR. Descriptive analyses were performed on multiple-choice polls and open-ended responses.

**Results:**

Polls received a mean of 317 responses (range, 222-504) and 20 open-ended comments. The most frequently cited student mistake was failure to understand their role in the CTOR (164 [32.5%]), followed by inappropriate chatting (141 [28.0%]) and breaking sterility (132 [26.2%]). Poll respondents valued students’ understanding of how to be helpful in the CTOR (101 [45.5%]) in addition to knowing what can or cannot be touched (47 [21.2%]) and basics of cardiopulmonary bypass (43 [19.4%]). Respondents indicated that students should assume active roles (84 [36.7%]), ask questions (66 [29%]), and help with minor tasks (41 [17.9%]). Respondents reported that students benefited most when they understood patient-specific clinicopathologic factors and basic operative steps.

**Conclusions:**

We identified several areas for student improvement in the CTOR. Development of educational resources addressing these issues may enhance interest and augment recruitment of students to cardiothoracic surgery.

**In Short**

▪ The skills and tasks essential to medical student success in the cardiothoracic operating room (CTOR) may not be evident to students, and educators must make intentional efforts to inform them of the expectations and culture of the CTOR.
▪ Resources regarding etiquette, behavioral norms, and expectations of medical students in the CTOR may enhance the learning experience and empower students to feel as though careers in cardiothoracic surgery are within reach.

Initial exposure to the cardiothoracic operating room (CTOR) may be overwhelming and isolating for medical students. The potential intimidation of the surgical environment combined with the complexity of cardiothoracic procedures may cause students to shy away from the field. Early exposure has been shown to have a significant impact on specialty choice.^1^ In addition, early mentorship fosters interest and retains students in the field^2^; however, it may not be available to all students. Limited resources exist for medical students rotating on cardiothoracic surgery services,^3^^,^^4^ but these tend to focus on patient care or on broad concepts of the operating room. Web-based educational resources for surgery are available, although they typically lack peer review and may be of variable quality.^5^ Cardiothoracic surgery would benefit greatly from enhancing opportunities for all medical students to gain exposure to the CTOR.

The primary objective of this study was to identify common knowledge gaps for medical students seeking to participate in the CTOR, with the eventual goal of developing an educational resource for medical students focusing on CTOR etiquette. As such, we aimed to query cardiothoracic surgeons, trainees, and operating room personnel to elucidate the common mistakes of students in the CTOR to develop a resource for medical students to optimize learning experiences.

## Material and Methods

We sought to gather perceptions from cardiothoracic surgeons, residents/fellows, anesthesiologists, operating room nurses, perfusionists, and surgical technologists on gaps and opportunities in medical student behavior in the CTOR. We developed a survey with multiple polls with topics regarding common experiences of CTOR personnel with medical students that also had potential for improvement. Whereas polls offered multiple-choice options, each poll also included an option for open-text commentary. This survey was first piloted among surgeons and CTOR personnel at a single institution, with feedback and adjustments made. The survey was subsequently distributed on social media outlets from June 27, 2022, through July 3, 2022.

The first topic investigated was the most common mistake medical students make in the CTOR (Supplemental Table). Prompted choices included breaking sterility, failing to understand their role, and inappropriate chatting. The second topic was the knowledge expectations of medical students in the CTOR. Prompted choices included the basics of cardiopulmonary bypass, what they can or cannot touch, and how to be helpful. The last topic investigated was what medical students should be prepared to do before entering the CTOR, with choices including placing Foley catheters and sequential compression devices, being active vs passive, and asking appropriate questions.

The social media platform X (formerly Twitter) was used as the method of distributing the survey to optimize the width of reach. Polling components remained open for 7 days, and data and comments were gathered after 9 days. We also gathered data on post views, engagements, likes, and reposts. Descriptive analyses were performed.

## Results

Each poll question received between 222 and 504 multiple-choice responses and in total 20 open-ended comments from self-identified cardiothoracic surgeons, anesthesiologists, residents, and perfusionists. Polls achieved a combined 31,145 social media engagements, 33 likes, 47 reposts, and 24 quotes/comments.

**Figure 1:**
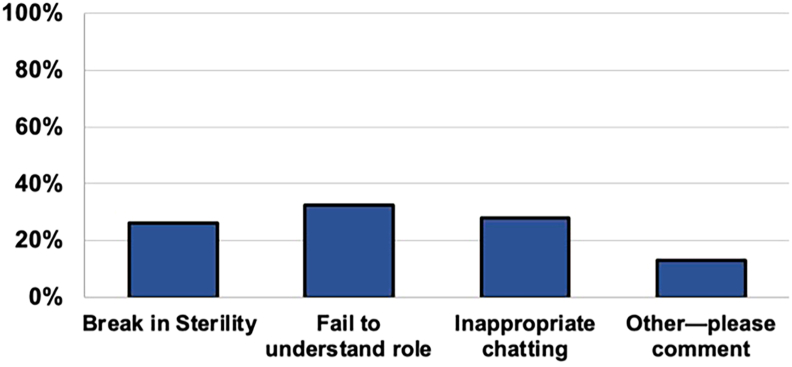
Responses regarding the most common mistake medical students make in the cardiothoracic operating room.

The most common medical student mistake reported by poll respondents was failure to understand their role in the CTOR (n = 164 [32.5%]). Inappropriate chatting (n = 141 [28.0%]) and breaking sterility (n = 132 [26.2%]) were also frequently cited (Figure 1). Thirteen percent of respondents provided additional free-text commentary, indicating deficits in preparation, proactivity, and situational awareness as additional common mistakes. Open-ended responses to all poll questions are detailed in the Table.

**Table:** Free-text Responses to Survey Questions Regarding Medical Student Behavior in the Cardiothoracic Operating Room

Question	Response
What is the most common mistake that you’ve seen medical students make in the CTOR?	Other: Haven’t read about patient or case
	An open educational environment with known expectations for our trainees enhances learning.
	Never step over the bypass lines. Go around the pump.
	Not proactively asking what they can do to help
	Situational awareness is key. Read the room. Sometimes it is best to take initiative. Sometimes it is best to watch/wait. You will get better at identifying this balance if you treat every person in the OR like a teacher.
	When you’re looking for a break in the case to ask your question, consider what the anesthesia team is doing too. If you are chatting while they are intubating, it’s dangerous and it conveys the message that you don’t understand that surgery is a team sport.
	Looking at these choices, it appears the issue is with students not being trained properly.
What would you want medical students to better understand before their first day in the CTOR?	Have them follow [educational social media pages].
	Come pay attention to what [anesthesiologists] are doing at the beginning of the case! We do a ton!
	Totally!!! I always tell students/residents to spend time with our anesthesia colleagues before a cardiac case gets going… arterial lines, swans, TEE … lots to learn!
If medical students could better prepare before CT surgery cases, what would you want them to be prepared to do?	Know the patient! Know the case! When I ask if anyone knows the PFTs…and the med student answers before the residents
	Know the patient and why we are doing the surgery. I think that’s a huge start! I always recommend to students to ask ahead how they can participate (what our student case goals are) and where they should stand during the beginning of the case when things can be chaotic!!
	Generically speaking, don’t be a passive learner: take your best shot at interpreting films (by viewing them, not just the report), reviewing the anatomy so that you can correlate what things look like IRL to the pics or videos, learning the steps of the operation and why, etc.
	Read around the case—the anatomy, the surgery (basics) and talk to the patient. And if they’re not learning anything useful and it’s midnight, go home!
	I think for new students, big CT operations can be very intimidating! When I was a third-year med student in the CT OR for the first time, I was terrified to touch anything! I slowly became more comfortable, but one of the things that helped me the most was a fellow who made an effort to tell me ways I could help (eg, Get scissors ready to cut when I start tying). I really wanted to participate and help, but I just didn’t know how until someone showed me some of the ways I could be helpful, and after that I was able to actively participate was much more helpful, felt more like a member of the team, and learned so much more!
	We ask students to prepare for 2-3 cases/wk—know patient history, disease, anatomy, and follow post-op. We do not expect to know the operation in detail. They are welcome to scrub other cases, but we consider it bonus.

CT, cardiothoracic; CTOR, cardiothoracic operating room; IRL, in real life; OR, operating room; PFT, pulmonary function test; TEE, transesophageal echocardiogram.

**Figure 2:**
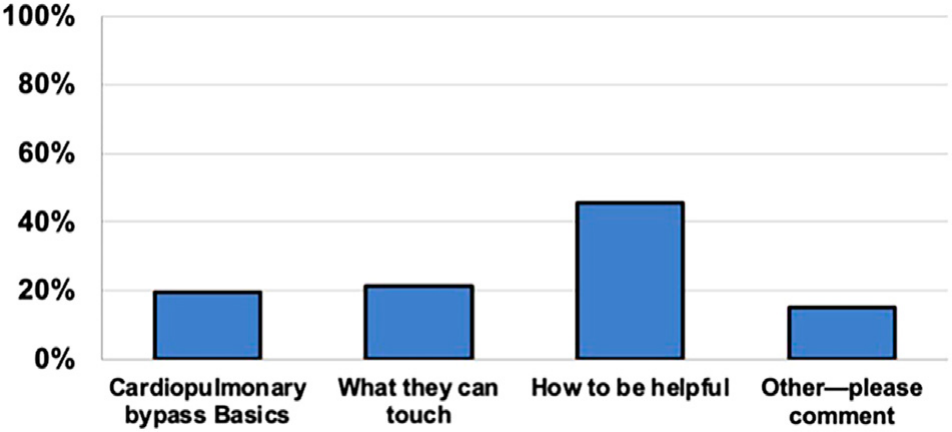
Responses regarding what respondents want medical students to better understand before their first day in the cardiothoracic operating room.

Poll respondents frequently indicated that medical students’ understanding of how to be helpful before entering the CTOR would be valuable (n = 101 [45.5%]). Respondents further indicated that knowing what can or cannot be touched (n = 47 [21.2%]) and the basics of cardiopulmonary bypass (n = 43 [19.4%]) would also be beneficial (Figure 2). Fifteen percent provided open comments, with a prevalent suggestion for students to engage with the anesthesia team for additional learning opportunities.

**Figure 3:**
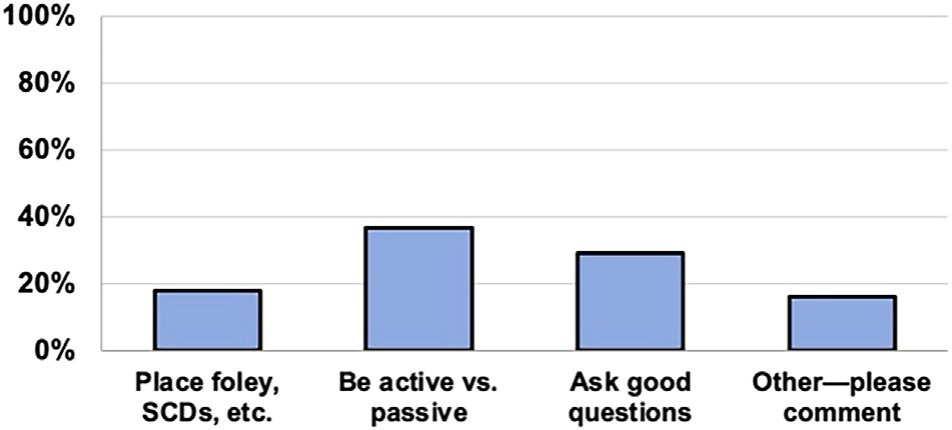
Responses regarding what medical students could better prepare for before cardiothoracic surgery cases. (SCDs, sequential compression devices.)

When asked what medical students should be prepared to do in the CTOR, poll respondents indicated that students should assume active roles in the CTOR rather than passively observing (n = 84 [36.7%]). Furthermore, medical students should be prepared to ask appropriate questions (n = 66 [29%]) and help with tasks such as placement of Foley catheters and sequential compression devices (n = 41 [17.9%]). Sixteen percent of respondents provided open comments on this topic (Figure 3). Respondents shared that students who understood patient-specific clinicopathologic factors benefited most from the CTOR. Knowing where to stand and how to be helpful was beneficial. Last, students who were aware of the basic steps of the case were better suited for educational CTOR experiences.

## Comment

This is the first investigation into appropriate and desirable medical student behavior in the CTOR. Through this research, several areas have been identified that can be targeted to improve medical student performance within the CTOR.

Entering the CTOR can be intimidating, and the role of the medical student is not always clear or properly communicated.^6^ Numerous studies have noted the importance of preparing medical students for the operating room, specifically preparation beyond technical skills and factual knowledge.^7^^,^^8^ However, most previous research focuses on factual knowledge and technical skills in the surgical sphere^3^^,^^4^ and resources regarding interpersonal, communication, and team-dynamic skills are lacking. Other surgical fields have developed curricula to prepare medical students for the operating room culture specific to their specialty, which increased student satisfaction with their operative and interpersonal experiences.^9^ Cardiothoracic surgery has yet to develop similar materials. Thus, the development of educational resources on the etiquette, behavioral norms, and expectations of medical students specific to the CTOR is necessary.

Using the results of this study, we have developed an online, freely accessible resource document for medical students entering the CTOR. We focused on the major areas of concern identified in this survey: understanding the medical student role, how to be helpful, and actively contributing to the team. Each section of the resource is written by a professional in that field to give students an understanding of how the CTOR functions and their place within it. We focus on professional expectations and social behavioral norms that can greatly shape a student’s experience. Honing these multidisciplinary viewpoints can decrease anxiety in the CTOR, which can ultimately improve their learning experience and positively inform their career decision-making.^6^ We hope this resource will provide comfort and guidance to all students entering the CTOR.

Limitations of this study are intrinsic to survey methods. Although the survey was distributed on a public platform, it is subject to selection bias, including limiting participants to those who use the platform. Poll responses were available only in aggregate, and characterization of respondents was not possible and therefore subject to responder bias. Multiple-choice options may limit the responses received, although respondents were encouraged to provide free text for additional input. We also recognize that the expectations of medical students may vary by institution and therefore may not be generalizable.

In conclusion, this study identified areas for medical student improvement in the CTOR and areas in which they can succeed and positively stand out. These skills and tasks may not be evident to students, and as educators, we must make intentional efforts to inform them of the expectations and culture of the CTOR. Furthermore, we must intentionally reach students who may not have opportunities for early cardiothoracic surgery mentorship to ensure equitable recruitment to the field. The resource developed from this study may enhance the student learning experience in the CTOR and guide all students so that they feel as though any specialty is within their reach.
