# Supplementary material for: Medical Student Role in the Cardiothoracic Operating Room: A Needs Assessment to Optimize Engagement
Source: Ann Thorac Surg Short Rep. 2024 Oct 16;3(1):276–80. doi: 10.1016/j.atssr.2024.09.017 (PMC11910795; doi:10.1016/j.atssr.2024.09.017)
Supplement: Supplementary Table 1 [file mmc1.docx]

| **Question** | **Answer Choices** |
| --- | --- |
| What is the most common mistake that you’ve seen medical students make in the cardiothoracic operating room? | Break in sterility |
|  | Fail to understand role |
|  | Inappropriate chatting |
|  | Other—please comment |
| What would you want medical students to better understand before their first day in the cardiothoracic operating room? | Cardiopulmonary bypass basics |
|  | What they can touch |
|  | How to be helpful |
|  | Other—please comment |
| If medical students could better prepare before cardiothoracic surgery cases, what would you want them to be prepared to do? | Place foley, SCDs, etc. |
|  | Be active vs. passive |
|  | Ask good questions |
|  | Other—please comment |
